# Genetic and functional variants of the *TBX20* gene promoter in dilated cardiomyopathy

**DOI:** 10.1002/mgg3.2355

**Published:** 2024-01-18

**Authors:** Xue Gao, Shuchao Pang, Liangcai Ding, Han Yan, Yinghua Cui, Bo Yan

**Affiliations:** ^1^ Cheeloo College of Medicine Shandong University Jinan Shandong China; ^2^ The Center for Molecular Genetics of Cardiovascular Diseases Affiliated Hospital of Jining Medical University, Jining Medical University Jining Shandong China; ^3^ Shandong Provincial Sino‐US Cooperation Research Center for Translational Medicine Affiliated Hospital of Jining Medical University, Jining Medical University Jining Shandong China; ^4^ Center for Molecular Medicine Yanzhou People's Hospital, Jining Medical University Jining Shandong China; ^5^ Division of Cardiology Affiliated Hospital of Jining Medical University, Jining Medical University Jining Shandong China; ^6^ Institute of Precision Medicine Jining Medical University Jining Shandong China

**Keywords:** dilated cardiomyopathy, promoter, single‐nucleotide polymorphisms, *TBX20*, variants

## Abstract

**Background:**

Dilated cardiomyopathy (DCM) is a major cause of heart failure and sudden cardiac death. As DCM is a genetically heterogeneous disease, genetic variants of cardiac transcription factor genes may play an important role. Transcription factor TBX20, an indispensable factor in normal heart development, is involved in the regulation of cardiac structure and function. Although the *TBX20* gene is associated with the occurrence and development of DCM, the influence of genetic variants of the *TBX20* gene promoter region on DCM has not been reported.

**Methods:**

We conducted a case–control study consisting of 107 DCM patients and 210 healthy controls. Genetic variants within *TBX20* gene promoter region were identified using sequencing techniques and were functionally analyzed by dual‐luciferase reporting assay. Electrophoretic mobility shift assay (EMSA) was used to investigate DNA‐protein interactions.

**Results:**

In this study cohort (*n* = 317), we identified eight variants within *TBX20* gene promoter. One novel DNA sequence variants (DSV) (g.4275G>T) and four single‐nucleotide polymorphisms (SNPs) [g.4169G>A (rs1263874255), g.4949C>T (rs1191745927), g.5114G>A (rs112076877), g.5252C>T (rs1356932911)] were identified in DCM patients, but in none of controls. Among them, the DSV (g.4275G>T) and three SNPs [g.4949C>T (rs1191745927), g.5114G>A (rs112076877) and g.5252C>T (rs1356932911)] significantly altered the transcription activity of *TBX20* gene promoter by dual‐luciferase reporting assay (*p* < 0.05). Further, EMSA assay indicated that the DSV (g.4275G>T) and three SNPs [g.4949C>T (rs1191745927), g.5114G>A (rs112076877) and g.5252C>T (rs1356932911)] affected the binding of transcription factors.

**Conclusions:**

These data indicate that the DSV (g.4275G>T) and three SNPs [g.4949C>T (rs1191745927), g.5114G>A (rs112076877) and g.5252C>T (rs1356932911)] increase transcription activity of *TBX20* gene promoter in both HEK‐293 and neonatal rat cardiomyocytes (NRCMs) cell lines by affecting the binding of transcription factors. But the mechanism remains to be verified in vivo.

## Graphical abstract

Variants in the TBX20 gene promoter significantly increased the transcriptional activity of the TBX20 gene promoter in HEK‐293 cell and NRCMs cell lines by affecting the binding of transcription factors.

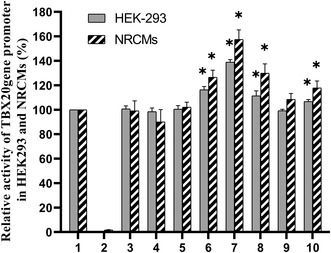

## 1 INTRODUCTION

Dilated cardiomyopathy (DCM) is one of the most common causes of heart failure, which is defined as the presence of LV dilatation and global or regional systolic dysfunction unexplained solely by abnormal loading conditions (e.g. hypertension, valve disease, CHD) or CAD (Arbelo et al., 2023). According to the classification of the cardiomyopathies of the European Society of Cardiology (ESC), DCM is grouped into familial and nonfamilial forms (Elliott et al., 2007). Familial DCM (FDCM) is inherited as autosomal dominant, X‐linked, autosomal‐recessive, and matrilinear modes of transmission in which autosomal dominant inheritance is present most commonly (Arbustini et al., 2000). Nonfamilial form is then classified into idiopathic (no identifiable cause) and acquired DCM (Elliott et al., 2007). Acquired DCM refers to ventricular dysfunction which is a complication of the disorder rather than an intrinsic feature of the disease. Acquired causes of DCM include nutritional deficiencies, infection, alcohol, administration of cardiotoxic drugs, etc (Elliott et al., 2007; Schultheiss et al., 2019). DCM is a complex and heterogeneous disease, whose genetic basis is diverse. Although with the development of sequencing technology, some variants have been discovered, which occurred in genes encoding a wide variety of proteins that are associated with sarcomere, cytoskeleton, sarcolemma, nucleus, the nuclear lamina, and Z‐disc (Jordan et al., 2021). The genetic causes in a significant fraction of the DCM cases have remained unknown.

TBX20 (OMIM: 606061), a member of the T‐box transcription factor (TBX) family, is conservatively expressed in the hearts of almost all species and involved in numerous cardiogenic processes and cardiac muscle differentiation by interacting with extensive transcriptional networks in different regions of developing hearts (Kraus et al., 2001; Meins et al., 2000). TBX20 directly interacts with NKX2‐5, GATA4 and GATA5 to regulate cardiac gene expression, and then coordinate cell proliferation, differentiation, and chamber formation (Brown et al., 2005; Stennard et al., 2003). Besides this, TBX20 can define the specification of chamber and non‐chamber myocardium, which provides a basis for cardiac morphogenesis through inhibiting TBX2‐mediated transcriptional function (Stennard et al., 2005). The research found *TBX20* knockout mice died at E10.5 with severe ventricular wall dysplasia and a defect heart looping (Singh et al., 2005). Therefore, TBX20 is essential for the normal heart development.

Previous reports discovered adult *TBX20* heterozygous knockout mice resulted in mild DCM without significant structural defects (Kirk et al., 2007). *TBX20* haploinsufficiency led to LV dilation, decreased wall thickness and contractile dysfunction, indicative of DCM (Stennard et al., 2005). In exception to this, *TBX20* may plays a role in the disease progression of DCM by altering expression levels. Research evidenced that *TBX20* expression was upregulated by measuring *TBX20* mRNA level in blood samples of IDCM patients and myocardium of rat model of DCM (Mittal et al., 2016). Vice versa, another research found overexpression of *TBX20* in myocardium of *TBX20* transgenic mice led to dilated cardiomyopathy that exhibited ventricular hypertrabeculation and an abnormal muscular septum (Zhang et al., 2011). It has been reported that although there is no variant in the gene itself, gene promoter variants also affect the binding of transcription factors, which can increase or decrease the genes expression, eventually leading to disease (Harakalova & Asselbergs, 2018). So far, the relationship between the variants of *TBX20* gene promoter and DCM has not been reported. Based on these studies, we speculated that the variants in *TBX20* gene promoter region may change *TBX20* gene expression and thus participate in the pathogenesis of DCM. To test this hypothesis, we designed a case–control study to genetically and functionally analyze the *TBX20* gene promoter in DCM patients.

## 2 MATERIALS AND METHODS

### 2.1 Study participants

Nonfamilial forms (sporadic) of DCM (SDCM) patients (*n* = 136) were recruited from the Division of Cardiology, Affiliated Hospital of Jining Medical University, Jining, Shandong, China. All DCM patients were diagnosed by medical history, clinical manifestations, physical examination, electrocardiograms, and echocardiography. Ethnically matched controls (*n* = 210, 136 males and 74 females) from the Physical Examination Center of the same hospital in the same period. Controls with DCM family history and other heart disease were excluded.

### 2.2 Direct DNA sequencing

Genomic DNA were extracted from peripheral leukocytes isolated from venous blood. As per methods described in our previous study, DNA fragments for *TBX20* gene promoter were generated by PCR and directly sequenced (Qiao et al., 2012). The design of PCR primers was based on the *TBX20* genome sequence (NCBI, Genbank: NG_015805.1), which covers translational start sites and the potential 5′ promoter region of *TBX20* gene. PCR primers were described in previous study (Qiao et al., 2012). DSVs were identified by comparing with wild‐type *TBX20* gene promoter. The effects of the DSVs in *TBX20* gene promoter on binding sites for transcription factors (TFs) were predicted by TRANSTAC database (https://portal.genexplain.com/).

### 2.3 Isolation of primary neonatal rat cardiomyocytes (NRCMs)

The research was approved by the animal Ethic Committee of the Affiliated Hospital of Jining Medical University (2021B080) and follows the 3R principle and the welfare principle of animal experiments. We have improved the protocols for the isolation of NRCMs, which form the past literature (Chen et al., 2020; Vandergriff et al., 2015). Neonatal rat pups (1–3 days old, gender unknown) were disinfected with 75% alcohol. Hearts were removed quickly while carefully removing any non‐heart tissue in PBS containing 1% penicillin–streptomycin. Then, hearts were cut into pieces in another new PBS containing 100 μ mL‐1 penicillin–streptomycin. Transferred tissue pieces to conical flask containing 3 mL 0.25% trypsin, rotated and digested near the alcohol lamp for 2 min, quickly aspirated and as much liquid as possible and discarded it. Repeated the step one time. Then, we added 3 mL 0.25% trypsin to the conical flask, rotated and digested near the alcohol lamp for 80 s, transferred the solution to the termination medium (14% FBS and 100 μ mL‐1 penicillin–streptomycin) to terminate digestion. Repeated digestion until the tissue was digested completely. The termination medium containing cell was transferred to a new centrifuge tube through the 70 μm cell strainer, then were centrifuged at 1000 rpm for 10 min and discarded the supernatant, resuspended the cell pellet in 10 mL low‐sugar medium (10% FBS and 100 μ mL‐1 penicillin–streptomycin) and transferred to petri dish. Incubated for 1.5 h at 37°C with 5% CO2. Next, transferred medium of petri dish to six‐well plate. The medium was changed with fresh medium after incubated for 24 h at 37°C with 5% CO2. The primary NRCMs were transfected after 36–48 h.

### 2.4 Functional analysis of regulatory variants by dual‐luciferase reporter

Primers containing NheI and XhoI digestion sites were designed according to the distribution of variants. [forward: 5′‐(NheI) CGACTGGCTGGAAAAGAGAACA‐3′; Reverse: 5′‐(XhoI) GGAGACAAAGACCCGAAACAC‐3′]. *TBX20* gene promoter (1448 bp, including wild type and mutant type) was generated by PCR, which were then inserted into the NheI and XhoI sites of a luciferase reporter vector (pGL3‐basic, Promega Corporation, Madison, WI, United States) to generate expression constructs [pGL3‐WT (wild type), PGL3‐4028C, PGL3‐4075T, PGL3‐4169A, PGL3‐4275T, PGL3‐4949T, PGL3‐5114A, PGL3‐5198A, PGL3‐5252T]. The Renilla luciferase vector (pRL‐TK) was used as reference reporter plasmid to control transcription efficiency and reduce the interference of changes of experimental conditions. The expression vector (pGL3 basic) without the *TBX20* gene promoter sequence was used as the negative control for transfection efficiency. Transcription activity of the *TBX20* gene promoter was tested in cell lines HEK‐293[CRL‐1573; American Type Culture Collection, Manassas, VA, United States (ATCC), Manassas, VA, United States] and NRCMs. Cells were seeded into 6‐well plates the day prior to lipofectamine transfection. The next day, 0.8 μg of the designated expression vector was transiently transfected into HEK293 and NRCMs. pRL‐TK were co‐transfected into HEK293 (50 ng) and NRCMs (100 ng) as internal controls. Fresh medium was replaced after 5 h. After 36 h, double luciferase activity was measured on a Glomax 20/20 luminometer (Promega Corporation, Madison, WI, United States) using Dual‐Luciferase®Reporter Assay according to the manufacturer's instructions. *TBX20* gene promoter activity was represented by dividing firefly luciferase activity by Renilla luciferase activity. Wild‐type *TBX20* gene promoter activity was set to 100%. Transfection experiments were repeated at least three times independently, three replicates per clone for each cell line. Cell lines of the whole experiment cultured at 37°C with 5% CO_2._

### 2.5 Nuclear extracts preparation and electrophoretic mobility shift assay

Electrophoretic mobility shift assay (EMSA) experiment was performed using the LightShift® Chemiluminescent EMSA kit (Thermo Fisher Scientific, Inc., Waltham, MA, United States) to examine the effects of *TBX20* gene regulatory variants on transcription factors binding sites (TFBS). Nuclear extracts were extracted from cultured HEK‐293 cells and NRCMs using NE‐PER®nuclear and cytoplasmic extraction reagents (Thermo Scientific, Rockford, IL). Protein concentrations were determined using the Bradford protein assay. Biotinylated double‐stranded oligonucleotide containing *TBX20* regulatory variants (30 bp) were used as probes (Table 1). First, pre‐electrophoresed the 6% polyacrylamide gel for 60 min in 0.5X TBE at voltage 100 V. We added the same amount of oligonucleotide probe (0.2 pmol), nuclear extract (3.0 μg), and other components supplied in the kit to 0.5 mL tubes according to the manufacturer's instructions. Total volume of each binding reaction was 20 μL. Incubated binding reactions at room temperature for 20 min. Next, each 20 μL binding reaction was gently mixed with 5 μL 5× loading buffer and loaded onto the 0.5 × TBE natural polyacrylamide gel. Samples were electrophoresed at voltage 100 V until the bromophenol blue dye has migrated approximately 2/3 to 3/4 down the length of the gel. Binding Reactions were transferred electrically to Nylon Membrane (Thermo Fisher Scientific, Inc., Waltham, MA, United States) at 380 mA (~100 V) for 30 min. Transmembrane solution was 0.5 × TBE. Then probes were cross‐linked to nylon membrane with the UV Stratalinker 1800 (Stratagene; Ailent Technologies, Inc., Santa Clara, CA, United States) and were detected by chemiluminescence.

**TABLE 1:** The double‐stranded biotinylated oligonucleotides for the EMSA.

Variants	Oligonucleotide sequences	Locations
g.4169 G>A	TTTGTAAAACCAGAG(G/A)GGTCTGTGTCCGCT	4154–4183
g.4275 G>T	CTTCGCCTTCTCTTC(G/T)GCAGCCGTCCCATT	4260–4289
g.4949 C>T	TGACTGGCTGCGGGC(C/T)TCCGGGATCGCCGC	4934–4963
g.5114 G>A	CCAGCCCCAGAGGGA(G/A)GAAGGACGCGGAGG	5099–5128
g.5252 C>T	TGCTCGGTGGGCCCT(C/T)TCCTCTCCGGCTGC	5237–5266

### 2.6 Statistical analysis

Transfection results were expressed by mean ± standard error of the mean and compared by a standard Student's *t*‐test and analyzed using two‐way analysis of variance followed by Dunnett test. The quantitative data were expressed as mean ± standard deviation and were analyzed by Student's *t*‐test. The qualitative data were analyzed using chi‐square test. The variants frequencies between DCM patients and controls were assessed using the Chi‐square test. Chi‐square test and student *t*‐test were performed with SPSS software 25.0 (SPSS, USA). *p* < 0.05 was considered as statistically significant.

## 3 RESULTS

### 3.1 Clinical and biochemical characteristics

We recruited 136 SDCM patients; 27 patients underwent coronary arteriography or CT coronary angiography according to symptom, in which 9 patients were considered coronary stenosis ≥50%, and 1 patient with myocardial bridge was considered coronary stenosis at 95% during systole. Based on the patient's history taking, physical examination, auxiliary examination, and family history, we excluded the patients with coronary heart disease (stenosis ≥50%), hypertension (non‐alcohol‐related hypertension), myocardial bridge, and congenital heart disease (CHD) according to the definitions of DCM of ESC consensus criteria. Sixty‐nine patients were deemed IDCM, 38 patients with acquired causes were deemed acquired DCM. Finally, the study included 317 participants (107 DCM patients and 210 control subjects), whose baseline clinical characteristics were shown in Table 2. The male proportion in the DCM group was significantly higher than that in the control group (*p* < 0.01), which may be related to the gender differences in DCM. There was no difference in the prevalence of hypertension and the age level between the two groups.

**TABLE 2:** The baseline clinical characteristics of the study subjects.

Variable	Control	DCM	*p*‐value
Coronary stenosis	0	14	–
Stenosis ≥50%	0	9	–
Hypertension (*n*, %)	59 (28.1%)	29 (21.3%)	–
Congenital heart disease	0	3	–
FDCM	0	0	–
IDCM	0	69	–
Acquired DCM	0	38	–
Age (years, mean ± SD)	45 ± 12	47 ± 13	0.062
Male (*n*, %)	136(64.8%)	95(88.8%)	<0.01
Diabetes (*n*, %)	20(9.5%)	13(12.1%)	0.469
Alcohol	–	33	–

Abbreviations: DCM, dilated cardiomyopathy; FDCM, family DCM; IDCM, idiopathic DCM.

### 3.2 Identified DSVs in the *TBX20* gene promoter

**FIGURE 1:**
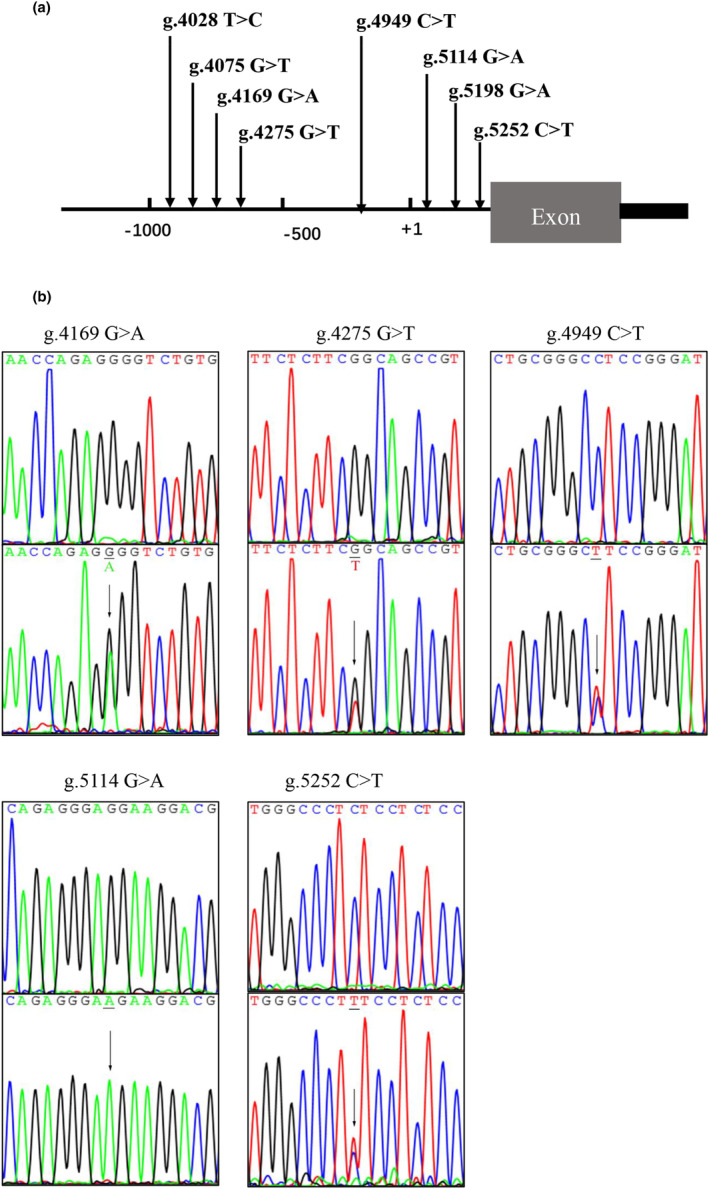
Regulatory variants of *TBX20* gene promoter. (a) Locations of *TBX20* gene promoter variants. Variants were named according to their locations in *TBX20* genomic sequences (NCBI, Genbank: NG_015805.1). The transcription starts at the position of 5001(+1) in the first exon. (b) Sequencing chromatograms of the variants identified in DCM patients. All sequence orientation of the DSVs and SNPs is forward. Top panels show wild‐type and bottom show mutant DNA sequences. Arrows indicate the variant sites.

A total of eight DSVs were found in this study population. Location distribution of the DSVs is shown in Figure 1a and their frequencies are summarized in Table 3. Among them, four SNPs [g.4169G>A (rs1263874255), g.4949C>T (rs1191745927), g.5114G>A (rs112076877), g.5252C>T (rs1356932911)] and one novel DSV (g.4275G>T) were only found in five DCM patients, but in none of the controls. The sequencing chromatograms of five DSVs are shown in (Figure 1b **)**. All five DCM patients were male, ranging in age at diagnosis from 43 to 60 years old. Clinical characteristics of five DCM patients were shown in Table 4. In addition, the SNP [g.5198G>A (rs139651523)] was identified in both DCM and controls with similar frequencies (*p* = 0.658). One SNP [g.4028T>C (rs186936022)] and one novel DSV (g.4075G>T) were only identified in the control group and their sequencing chromatograms were not shown.

**TABLE 3:** Variants of *TBX20* gene in DCM patients and controls.

DSVs	Genotypes	Location	Clinical significance	MAF^a^	Controls (*n* = 210)	DCM (*n* = 107)	*p*‐value
g.4028T>C (rs186936022)	TC	−973 bp	Not reported	0.0009330	2	0	–
g.4075G>T	GT	−926 bp	–	–	1	0	–
g.4169G>A (rs1263874255)	GA	−832 bp	Not reported	0.00001314	0	1	–
g.4275G>T	GT	−726 bp	–	–	0	1	–
g.4949C>T (rs1191745927)	CT	−52 bp	Not reported	0.00001974	0	1	–
g.5114G>A (rs112076877)	GA	+113 bp	Likely‐benign	0.003706	0	1	–
g.5198G>A (rs139651523)	GA	+197 bp	Not reported	0.001256	23	10	0.658
g.5252C>T (rs1356932911)	CT	+251 bp	Not reported	0.00001314	0	1	–
Total					26	15	0.681

*Note*: Variants are located upstream (−) or downstream (+) to the transcription start site of the *TBX20* gene at position 5001 (NG_015805.1). ATG (translation start site) is within the first exon and at the position 5481 of NG_015805.1. DSVs: DNA sequence variants; MAF, minor allele frequency.

^a^ The data comes from ganomAD database (ganomAD v3.1.1).

**TABLE 4:** Clinical characteristics of the DCM patients carrying *TBX20* gene variants.

Patient no.	1	2	3	4	5
Regulatory variant	g.4169G>A	g.4275G>T	g.4949C>T	g.5114G>A	g.5252C>T
Sex	M	M	M	M	M
Age (diagnosis)	47	44	51	60	43
LVEF (%)	16	24	21	26	35
LVEDD (mm)	70	80	70	79	67
NYHA	IV	IV	IV	III‐IV	III‐IV
Hypertension	No	No	No	No	No
Diabetes	No	No	No	Yes	No
Alcohol	No	No	Yes	No	No
Coronary heart disease	No	No	No	No	No
Congenital heart disease	No	No	No	No	No

### 3.3 Functional analysis of the DSVs by dual‐luciferase reporter assay

We used a dual‐luciferase reporter to determine the effect of variants on the transcriptional activity of *TBX20* gene promoter. Expression vector were co‐transfected with PRL‐TK (internal reference) into HEK‐293 cells and NRCMs, respectively. After 36 h, the dual‐luciferase activities were measured, and relative activity of wild‐type and variant *TBX20* gene promoters was examined. The pRL‐TK and empty vector (pGL3‐basic) were used as internal and negative control, respectively.

**FIGURE 2:**
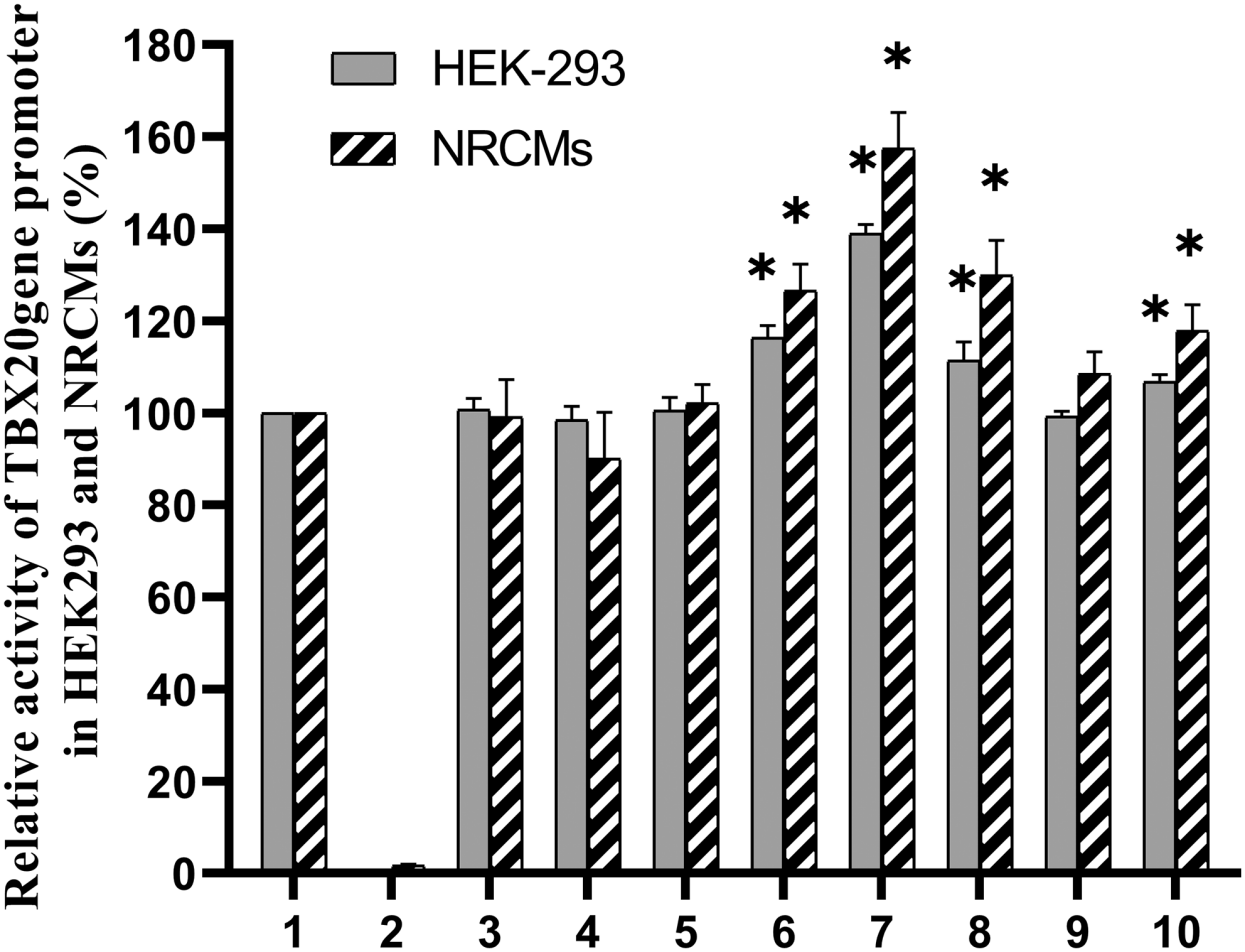
Relative transcriptional activity of wild‐type and variant *TBX20* gene promoters in HEK‐293 cells and NRCMs. Empty vector pGL3‐basic was used as a negative control. Transcriptional activity of the wild‐type *TBX20* gene promoter was designed as 100%. Relative activity of variant *TBX20* gene promoters was calculated. Lanes 1, pGL3‐WT; 2, pGL3‐basic; 3, PGL3‐4028C; 4, PGL3‐4075T; 5, PGL3‐4169A; 6, PGL3‐4275T; 7, PGL3‐4949T; 8, PGL3‐5114A; 9, PGL3‐5198A; 10, PGL3‐5252T. *p < 0.05. NRCMs, Neonatal Rat Cardiomyocytes.

In HEK‐293 cells, three SNPs (g.4949C>T, g.5114G>A, g.5252C>T) and one DSV (g.4275G>T) significantly enhanced the transcriptional activity of *TBX20* gene promoter (*p* < 0.05). The numeric magnitude of the increase in transcriptional activity was 38.92% (g.4949T), 13.89% (g.5114A), 7.85% (g.5252T), 16.33% (g.4275T). The above results indicated that the transcriptional activity of *TBX20* gene promoter was changed by the four variants. None of the other variants identified in the study altered the transcriptional activity of the *TBX20* gene promoter (*p* > 0.05) (Figure 2). As human cardiomyocyte cell lines are currently not available, primary NRCMs are used. Similar results to that in HEK‐293 cells were obtained in primary NRCMs. Three SNPs (g.4949C>T, g.5114G>A, g.5252C>T) and one DSV (g.4275G>T) significantly increased the transcriptional activity of *TBX20* gene promoter (*p* < 0.05) (Figure 2). The numeric magnitude of the increase in transcriptional activity was 57.46% (g.4949T), 29.90% (g.5114A), 17.86% (g.5252T), 21.63% (g.4275T).

### 3.4 Transcription factor binding as determined by EMSA

**FIGURE 3:**
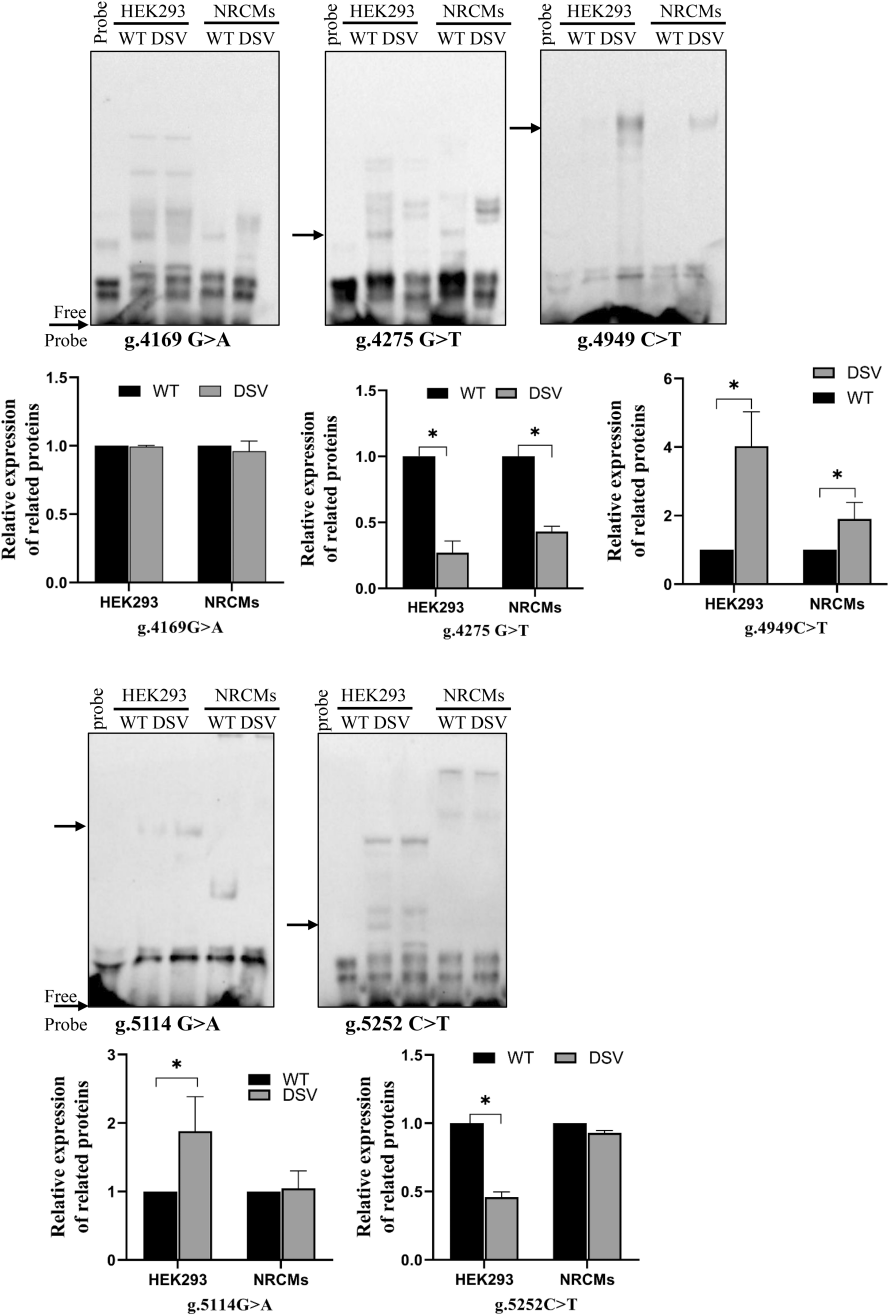
EMSA of biotin‐labeled oligonucleotide‐containing variants with nuclear extracts of HEK‐293 and NRCMs. The free probe was marked at the bottom. The affected binding for an unknown transcription factor was marked with an arrow. DSV, DNA sequence variants; NRCMs, neonatal rat cardiomyocytes; WT, wild type.

Wild‐type and variant oligonucleotide probes (30 bp) were designed for EMSA assay to verify whether the variant site affected the binding of transcription factors. EMSA results are shown in Figure 3. We found the DSV (g.4275G>T) abolished the binding site of unknown transcription factor. The SNP (g.4949C>T) created the binding site of unknown transcription factors. The SNP (g.5114G>A) enhanced the binding site of unknown transcription factor. The SNP (g.5252C>T) may abolish the binding site of unknown transcription factors. While the SNP (g.4169G>A) did not affect the binding site of transcription factors.

### 3.5 DSVS affected binding sites of transcription factors

The key to the regulation of gene expression lies in the regulation of the transcription initiation level, so it is important to study the effects of *TBX20* gene promoter variants on transcription factors. The TRANSFAC database is the most comprehensive database for collecting information on transcription factors and transcription factor binding sites. To determine whether DSVs affect putative biding sites for transcription factors, the *TBX20* gene promoter was analyzed with TRANSFAC database. The DSVs identified in DCM patients may abolish, create, and modify the putative binding sites for transcription factors. We found that the DSVs in the promoter region of *TBX20* may change the binding of the transcription factors by searching TRANSFAC database. As indicated in Table 5, the DSV (g.4275G>T) may abolish the binding site of TFIIB and SMAD1; and create the binding sites of SMAD5. The SNP (g.4949C>T) create the binding sites of ELF2, ERG, and GABP‐alpha; modified the binding sites of CPBP. The SNP (g.5114G>A) abolish the binding sites of GKLF and PU.1; create the binding sites of ZNF35. The SNP (g.5252C>T) disrupt the binding sites of CSX and produced the binding sites of NFAT and EHF.

**TABLE 5:** Predicted binding sites for transcription factors affected by the DSVs.

DSVs	Predicted change	Transcription factors
g.4275G>T	Abolish	TFIIB, SMAD1
	Create	SMAD5
g.4949C>T	Create	ELF2, ERG, GABP‐alpha
	Modify	CPBP
g.5114G>A	Create	ZNF35
	Abolish	GKLF, PU.1
g.5252C>T	Create	NFAT, EHF
	Abolish	CSX

Abbreviations: CPBP, Core Promoter Element‐Binding Protein; CSX, Cardiac‐Specific Homeobox; EHF, ETS Homologous Factor; ELF2, E74‐Like Factor 2; ERG, ETS Transcription Factor ERG; GABP‐alpha, GA‐Binding Protein Alpha; GKLF, Gut Kruppel‐Like Factor; NFAT, Nuclear Factor of Activated T‐Cells; PU.1, Transcription Factor PU.1; SMAD1, SMAD family member 1; SMAD5, SMAD family member 5; TFIIB, transcription initiation factor IIB; ZNF35, Zinc Finger Protein 35.

## 4 DISCUSSION

DCM commonly results from interactions between genetic and environmental factors, the causes of which are complex and heterogeneous. It is important to note that all the etiologies are not mutually exclusive and may occur in combination. 2023 ESC Guidelines for the management of cardiomyopathies indicated that cardiomyopathies can coexist with ischemic, valvular, and hypertensive disease and that the presence of one does not exclude the possibility of the other (Arbelo et al., 2023). Evidence suggests that genetic predisposition in DCM may interact with extrinsic disease triggers such as toxin exposure (ethanol, chemotherapy, and cocaine), viral infection, and pregnancy (Asselbergs et al., 2020). Patients who are carriers of pathogenic variants may also suffer episodes of myocarditis or report excessive ethanol intake, which may sequentially aggravate their overall clinical picture (Orphanou et al., 2021).

In this study, five variants (g.4275T, g.4949T, g.5114A, g.5252T) in *TBX20* gene promoter were detected in five DCM patients, accounting for 4.7% (5/107) of DCM‐affected patients screened. Furthermore, we found the MAF (minor allele frequency) of these SNVs were very low by searching gnomAD database. It is regrettable that clinical significance and phenotype of most SNVs were not reported beside g.5114G>A. The clinical significance associated with g.5114G>A is likely benign, and the clinical phenotype of it was not specified. To determine the function characteristics of five variants in DCM patients, to evaluate the effect of variants on *TBX20* gene. We observed whether variants alter *TBX20* gene promoter activity by functional analysis. Although the trend of *TBX20* gene promoter activity was the same in both cell lines, the numeric magnitude of *TBX20* gene promoter activity in primary NRCMs was higher than that in HEK‐293 cells. The difference in the degree of influence may be due to tissue‐specific expression. Surprisingly, SNP (g.4169G>A) found in DCM patients did not significantly change *TBX20* gene promoter transcriptional activity (*p* > 0.05), indicating that not all promoter sequence variants affect transcriptional regulation.

The increasing *TBX20* gene promoter transcriptional activity may promote *TBX20* overexpression. Research confirmed that *TBX20* overexpression induces activation of BMP2/pSmad1/5/8 and PI3K/AKT/GSK3β/β‐catenin signaling concomitant with increased cell proliferation (Chakraborty et al., 2013). Besides, ISL1 transcription factors are essential for embryonic cardiogenesis and postnatal cardiac remodeling, which is related to DCM (Xu et al., 2019). TBX20 directly binds to the conserved T‐half sites within the ISL1 promoter sequence and negatively regulates ISL1 expression (Cai et al., 2005). TBX2 is a ventricular inhibitory factor. TBX20 can directly weaken the BMP/Smad signaling, thereby inhibit *TBX2* expression in chamber, and indirectly restrict precocious *TBX2* transcription by isolating BMP‐activated Smad1/5/8 (Singh et al., 2009). In conclusion, the enhanced *TBX20* gene transcriptional activity may affect the activation of cardiac genes/signaling pathways, contributing to DCM. However, *TBX20* haploinsufficiency also led to DCM, which might have disturbed the cardiac transcription factor network. This is because *TBX20* heterozygous mutants commonly resulted in the expression change of genes related to heart development.

The most important reason for the disease caused by promoter sequence variant is that transcriptional regulation disorder results from creation or destruction of TFBSs, which disrupts normal gene transcription and activation. Therefore, promoter variants may decrease or increase the level of mRNA and protein (de Vooght et al., 2009). In fact, EMSA experiments revealed that the DSV (g.4275G>T) and SNPs (g.4949C>T, g.5114G>A, g.5252C>T) affect the binding affinity of transcription factors. Among them, the SNP (g.5114G>A) disrupted the binding of transcription factors in HEK‐293 cells, but had no effect on NRCMs cells. The SNP (g.5252C>T) disrupted the binding of transcription factors in NRCMs cells, but had no effect on HEK‐293 cells, which may be attributed to the specificity of tissue cells or the sensitivity of EMSA test.

*TBX20* expression is strictly regulated by transcription factors. It has been demonstrated that the promoter element is both necessary and sufficient to drive cardiac‐specific expression of *TBX20* in experimental animals (Mandel et al., 2010). In BMP/Smad1 signaling pathway, phosphorylated Smad1 directly binds to novel, non‐canonical, high‐affinity Smad1 sites to activate *TBX20* expression, which is necessary for *TBX20* expression in Xenopus (Mandel et al., 2010). In vitro promoter analysis proved that BMP10 could also induce *TBX20* promoter activity through a conserved Smad binding site in the *TBX20* promoter proximal region, and removal of BMP10 reduced *TBX20* expression specifically of in the BMP10 expression region of the developing ventricular (Zhang et al., 2011). In addition, predictive sites of specific transcription factors, such as SP1, ETS, CREB and RFX, have also been found to be associated with high promoter activity (Andersson & Sandelin, 2020; Nguyen et al., 2016). Combined with the results of cell transfection, TRANSFAC prediction, and EMSA, we extrapolated that the DSV (g.4275G>T) may abolish the binding site of smad1(SMAD family member 1), and produce the binding sites of smad5(SMAD family member 5). The SNP (g.4949C>T) generate the binding sites of ELF2(E74‐Like Factor 2) and ERG (ETS Transcription Factor ERG). The SNP (g.5114G>A) disrupt the binding sites of GKLF (Gut Kruppel‐Like Factor) and PU.1 (Transcription Factor PU.1) and create the binding sites of ZNF35(Zinc Finger Protein 35). The SNP (g.5252C>T) disrupt the binding sites of CSX (Cardiac‐Specific Homeobox) and produced the binding sites of EHF (ETS Homologous Factor) and NFAT (Nuclear Factor of Activated T‐Cells).

This study has some limitations. First, the sample size used in this study was relatively small. Five variants found in DCM patients only appeared in one patient, respectively. And they did not show any statistical significance in statistical analysis. Secondly, although reporter gene analysis is the most accurate method available to investigate the functional consequences of promoter variants, due to differences in chromatin environment, the activity of reporter genes in vivo may fail to reproduce the expression patterns of their endogenous equivalents. Therefore, it is necessary to further confirm the discovery in animals. Finally, the quality of the data used to build the TFBS matrix of TRANSFAC database will lead to the false‐positive problems and the false‐negative problems caused by the incomplete DNA binding factor in the database (de Vooght et al., 2009). Therefore, it is necessary to further identify the transcription factors of TRANSFAC prediction.

In conclusion, the DSV (g.4275G>T) and SNPs (g.4949C>T, g.5114G>A, g.5252C>T) within *TBX20* gene promoter region significantly increased the transcriptional activity of *TBX20* gene promoter in HEK‐293 cell and NRCMs cell by affecting the binding of transcription factors. Whether the four variants identified within the *TBX20* gene promoter can promote the development and progression of human DCM by altering *TBX20* expression levels still requires further studies to confirm.

## AUTHOR CONTRIBUTIONS

Bo Yan designed the study. Xue Gao, Shuchao Pang, Liangcai Ding, and Han Yan collected the samples and performed the experiments. Xue Gao, Yinghua Cui analyzed the data. Bo Yan and Xue Gao wrote, reviewed, and edited the article. All authors read and approved the final article.

## FUNDING INFORMATION

This study was supported by National Natural Science Foundation of China (81870279).

## CONFLICT OF INTEREST STATEMENT

The authors declare no conflict of interest.

## ETHICS STATEMENT

This study was approved by the Human Ethics Committee of the Affiliated Hospital of Jining Medical College (2018‐FY‐070) and was carried out in adherence to the principles of the Helsinki Declaration (1964). All participants provided written informed consent.

## Supporting information


Supporting information

## Data Availability

The data presented in this study are available in the article and supplementary material.
