# Supplementary figures and images for: Genetic and functional variants of the TBX20 gene promoter in dilated cardiomyopathy
Source: Mol Genet Genomic Med. 2024 Jan 18;12(1):e2355. doi: 10.1002/mgg3.2355 (PMC10795084; doi:10.1002/mgg3.2355)

Sequencing chromatograms of variants that weren’t shown in figure 1(B)

g.4028 T>C g.4075 G>T g.5198 G>A


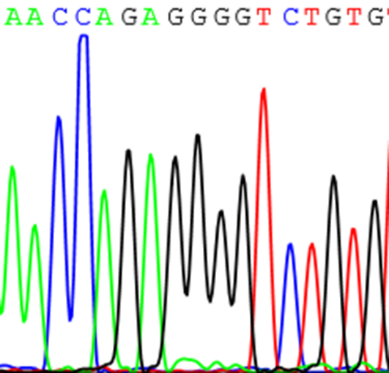

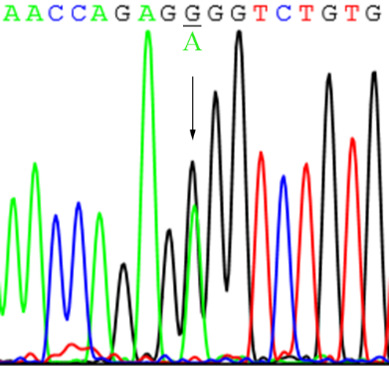

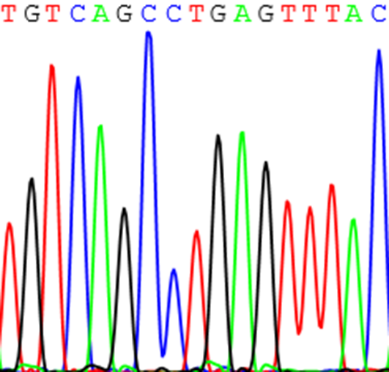

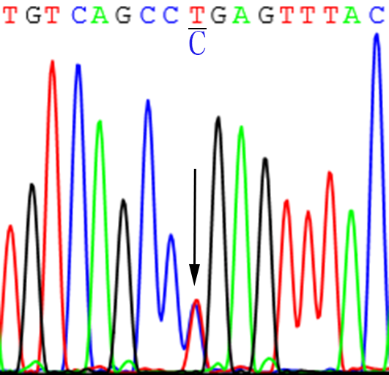

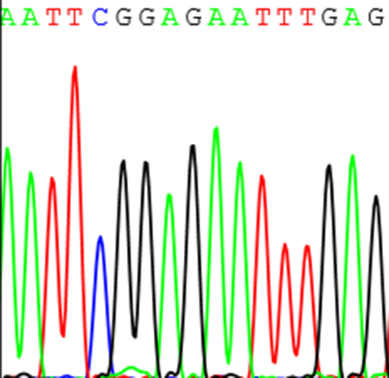

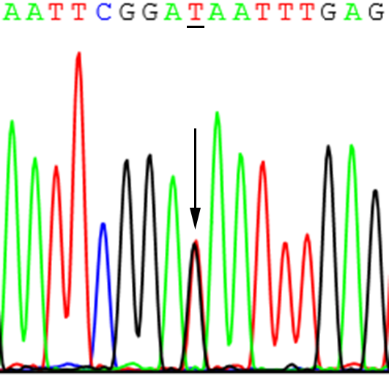

Supplement: Supplementary file 1 — Supporting information [file MGG3-12-e2355-s001.zip › Supporting information/suppoting information 2.docx]
